# A database to enable discovery and design of piezoelectric materials

**DOI:** 10.1038/sdata.2015.53

**Published:** 2015-09-29

**Authors:** Maarten de Jong, Wei Chen, Henry Geerlings, Mark Asta, Kristin Aslaug Persson

**Affiliations:** 1 Department of Materials Science and Engineering, University of California, Berkeley, California 94720, USA; 2 Environmental Energy Technologies Division, Lawrence Berkeley National Laboratory, Berkeley, California 94720, USA; 3 Department of Mechanical, Materials and Aerospace Engineering, Illinois Institute of Technology, Berkeley, Chicago IL 60616, USA

**Keywords:** Actuators, Electronic structure, Computational methods

## Abstract

Piezoelectric materials are used in numerous applications requiring a coupling between electrical fields and mechanical strain. Despite the technological importance of this class of materials, for only a small fraction of all inorganic compounds which display compatible crystallographic symmetry, has piezoelectricity been characterized experimentally or computationally. In this work we employ first-principles calculations based on density functional perturbation theory to compute the piezoelectric tensors for nearly a thousand compounds, thereby increasing the available data for this property by more than an order of magnitude. The results are compared to select experimental data to establish the accuracy of the calculated properties. The details of the calculations are also presented, along with a description of the format of the database developed to make these computational results publicly available. In addition, the ways in which the database can be accessed and applied in materials development efforts are described.

## Background & Summary

Piezoelectricity is a reversible physical process that occurs in some materials whereby an electric dipole moment is generated upon the application of a stress. This is often referred to as the direct piezoelectric effect. Conversely, the indirect piezoelectric effect refers to the case when a strain is generated in a material upon the application of an electric field^1^. Today, piezoelectric materials are integral to numerous applications and devices that exploit this effect, and form the basis for a multi-billion dollar worldwide market^2,3^. Examples are found in high voltage and power applications, actuators, sensors, motors, atomic force microscopes, energy harvesting devices and medical applications. These technologies all rely on the conversion of voltage to mechanical deformation or vice versa.

**Figure 1:**
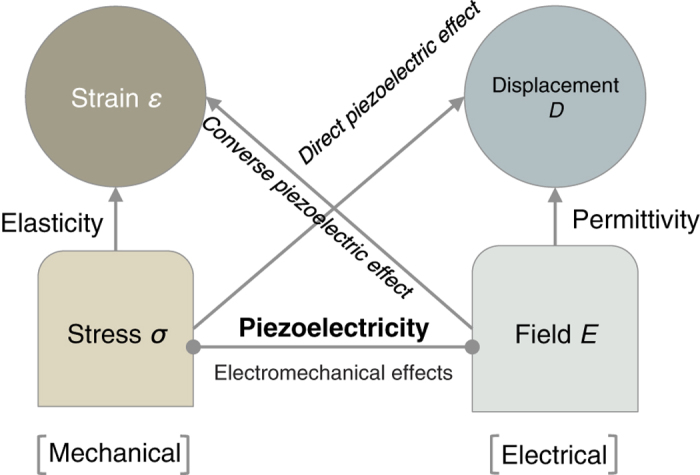
A Heckmann diagram. Part of a Heckmann diagram, showing the relation between mechanical and electrical properties of solids. After Nye^1^.

The mathematical description of piezoelectricity relates the strain (or stress) to the electric field via a third order tensor. This tensor describes the response of any piezoelectric bulk material, when subjected to an electric field or a mechanical load. The Heckmann diagram (Fig. 1)^1^ conveniently describes how mechanical and electrical properties of solids are related. The piezoelectric response of a material can be described using different piezoelectric constants, reflecting various derivatives of thermodynamic functions. Of particular interest to this work are the isothermal piezoelectric stress constants (abbreviated in the rest of this paper as simply piezoelectric constants), defined in full tensor notation as $e_{ijk}^{T}={\left(\frac{\partial D_{i}}{\partial \varepsilon_{jk}}\right)}_{E,T}=-{\left(\frac{\partial \sigma_{jk}}{\partial E_{i}}\right)}_{\varepsilon ,T}$, where *D*, *E*, *ε*, *σ* and *T* represent the electric displacement field, the electric field, the strain tensor, the stress tensor and the temperature, respectively. In this work, Voigt-notation is employed for brevity so that the relations for the piezoelectric constants read $e_{ij}^{T}={\left(\frac{\partial D_{i}}{\partial \varepsilon_{j}}\right)}_{E,T}=-{\left(\frac{\partial \sigma_{j}}{\partial E_{i}}\right)}_{\varepsilon ,T}$. The Voigt-notation will be explained below in more detail. We note that the most commonly used piezoelectric constants appearing in the (experimental) literature are the piezoelectric strain constants, usually denoted by *d*_*ijk*_. These can be readily related to the constants *e*_*ijk*_ if the elastic compliances $s_{lmjk}^{ET}$ (at constant electric field and temperature) of the materials are known^4^: $d_{ijk}^{T}=e_{ilm}s_{lmjk}^{ET}$. In particular, the piezoelectric strain constants can be expressed thermodynamically as^1^ $d_{kij}^{T}={\left(\frac{\partial \varepsilon_{ij}}{\partial E_{k}}\right)}_{\sigma ,T}={\left(\frac{\partial D_{k}}{\partial \sigma_{ij}}\right)}_{E,T}$.

The complete piezoelectric tensor has been measured or calculated for only a small subset of potential piezoelectric materials. In the main references of compiled materials properties, a total of less than 50 systems can be found for which experimental and/or calculated values for full piezoelectric tensors are available^5–22^. This represents a small subset of candidate materials, since in principle all materials with a finite bandgap that lack inversion symmetry can exhibit piezoelectric behavior. Hence there are thousands of hitherto unknown potential piezoelectric compounds in the inorganic crystal structure database^23–25^. Recently, significant effort has been devoted to the development of lead-free piezoelectric materials^26–32^. However, efficient screening over a wide range of materials chemistries is hindered by the lack of comprehensive experimental data.

Another challenge associated with the available data for piezoelectric constants is the large variability in the reported values, depending on the details of the experimental or computational techniques employed and/or the conditions under which the experiments are performed. As an example, *α*-quartz (SiO_2_) is the second most abundant mineral and a commonly employed material in piezoelectric devices. However, its reported piezoelectric constants differ by up to a factor of 3 in magnitude, presumably depending on the experimental conditions^5^ and temperature^14,33^. The same is true for the common mineral AlPO_4_ and elemental Te^5^.

In this paper, we introduce the to-date largest database of consistently calculated piezoelectric tensor properties of dielectric crystalline inorganic compounds. This database supplements our earlier work on elastic constants^34–38^ and contributes to a more complete description of the deformation behavior of solids. Whereas our previous work was limited to describing the relationship between stress and strain in the absence of electric fields, we now introduce the piezoelectric constants to incorporate this effect. Based on the Heckmann diagram in Fig. 1, this addition of piezoelectric constants will significantly increase the applicability of our previous dataset comprising zero-electric field elastic constants^34^.

The work presented in this paper is part of the Materials Project (MP) (www.materialsproject.org)^39^, and aims at employing high-throughput (HT) methods^40,41^ to develop open databases of calculated materials properties for discovery and design. The database of piezoelectric tensors currently consists of 941 materials and efforts are underway to compute more compounds in the near future. The piezoelectric properties are obtained using first-principles quantum-mechanical calculations based on Density Functional Theory (DFT), in particular Density Functional Perturbation Theory (DFPT)^42–44^. As described below, the calculated piezoelectric constants show a level of agreement with experimental data that is often comparable to the scatter in the measured data itself. It is important to note that in this paper, intrinsic piezoelectric constants are presented, associated with the bulk, defect-free and strain-free material, at a temperature of 0 K.

The remainder of the paper is organized as follows. In the next section the methods for materials selection and calculation of piezoelectric constants within an HT approach are described. Subsequently, the results of verification and validation analyses are presented, which establishes the accuracy of our DFT-calculations as well as the HT-algorithms. Finally, the last part of the paper describes the structure of the data and gives an overview of the results obtained in this study.

## Methods

### Definitions & computational settings

In this work we report calculated values for the proper piezoelectric constants, *e*_*ij*_, defined as follows:

(1)eijT=−(∂σj∂Ei)ε,T,j↦\{11,22,33,12,23,31\},i↦\{1,2,3\}

where *σ* denotes the stress tensor and *E* denotes the electric field. Also, in the Voigt notation used in this work, pairs of Cartesian directions are contracted as follows: 11↦1, 22↦2, 33↦3, 23, 32↦4, 13, 21↦5, 12, 21↦6. The piezoelectric stress is the sum of the ionic and electronic contributions and the piezoelectric tensor-components as defined in equation (1) have units C/*m*^2^.

**Figure 2:**
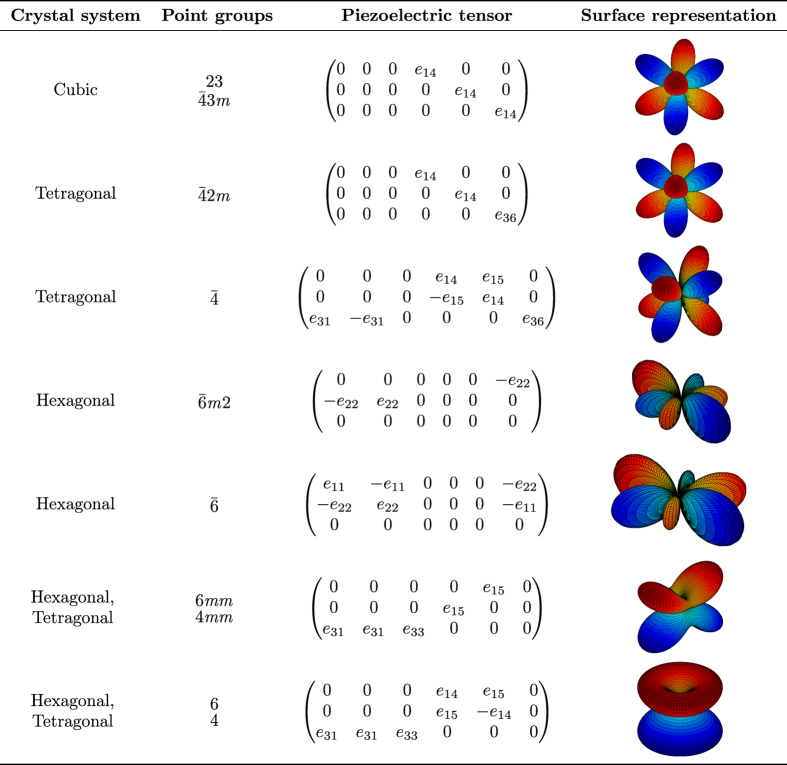
An overview of the symmetry constraints on piezoelectric tensors for various crystal point groups, together with surface representations of the piezoelectric tensor. Piezoelectric tensors and symmetry classes considered in this work, part I. Typical representations of the longitudinal piezoelectric modulus in 3D are also shown for each crystal point group.

**Figure 3:**
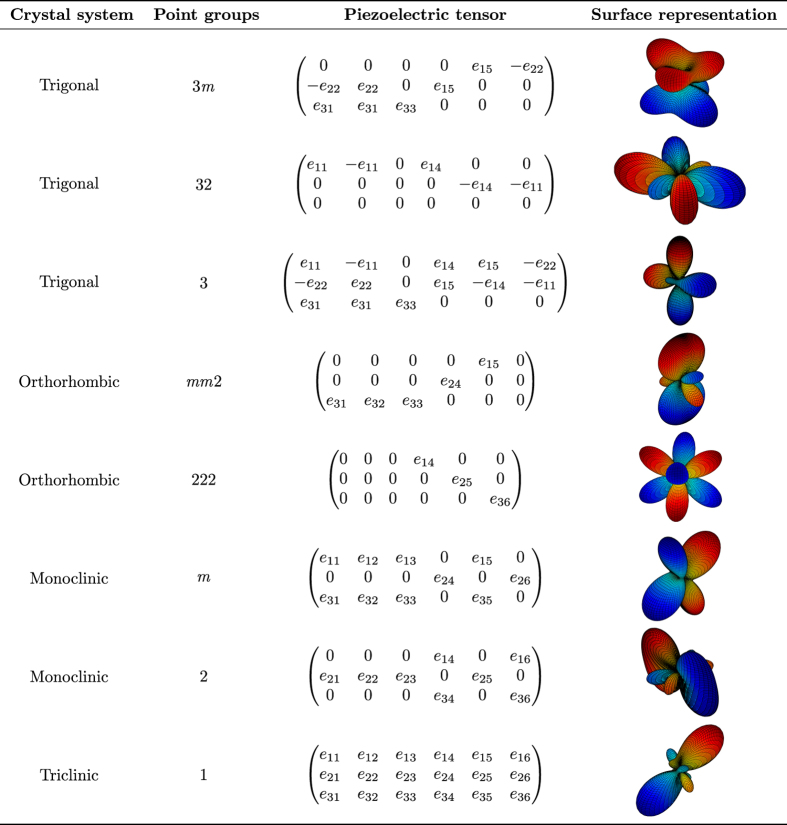
An overview of the symmetry constraints on piezoelectric tensors for various crystal point groups, together with surface representations of the piezoelectric tensor. Piezoelectric tensors and symmetry classes considered in this work, part II. Typical representations of the longitudinal piezoelectric modulus in 3D are also shown for each crystal point group.

Note that different matrix-tensor notations exist in the literature. In particular, when mapping the full third order piezoelectric tensor onto a 3×6-matrix, factors of 2 pre-multiplying certain constants are sometimes introduced. Specifically, some authors use the convention that *e*_*ijk*_=*e*_*in*_, when *n*=1, 2, 3 and 2*e*_*ijk*_=*e*_*in*_, when *n*=4, 5, 6 (refs 1,12,45–47). In the present work, factors of 2 and $\frac{1}{2}$ are not introduced in the piezoelectric tensor itself, but rather in the vectors operating on the piezoelectric tensor. Figures 2 and 3 (discussed in detail below) indicate for different crystal systems and point groups, the form the piezoelectric tensors take, according to the notation employed in this work. It is straightforward to convert to other conventions found in the literature.

The first-principles results presented in this work are performed using the projector augmented wave (PAW) method^48,49^ as implemented in the Vienna Ab Initio Simulation Package (VASP)^50,51^. In all calculations, we employ the Perdew, Becke and Ernzerhof (PBE) Generalized Gradient Approximation (GGA) for the exchange-correlation functional^52^. A cut-off for the plane waves of 1000 eV is used and a uniform k-point density of approximately 2,000 per reciprocal atom (pra) is employed, which means that the number of atoms per cell multiplied by the number of k-points equals approximately 2,000. For the compounds that contain magnetic elements, a ferromagnetic state is initialized in the calculation. Similarly to our previous work^34^, we expect to correctly converge to ferromagnetic and non-magnetic states in this way, but not to anti-ferromagnetic states. Due to the presence of strongly correlated electrons in some of the oxides, the GGA+U method is employed, with U representing the Hubbard-parameter^53,54^. The values of U are chosen consistent with those employed in MP^39,55^.

We estimate that the choice for plane wave cutoff and kpoints leads to piezoelectric tensors with components that are converged to within approximately 10% for over 90% of the considered systems. This is based on careful convergence testing of DFT-results on a representative subset of approximately 25 compounds^5–20^. Given the large variety of compounds and elements considered in this work, our DFT-parameters cannot be expected to perform equally well for all systems under investigation. Therefore, consistency checks are devised in our HT-infrastructure to detect possible anomalous behavior and errors in the first-principles calculations, similar to those devised for the HT-calculations of the recently published elastic constants^34^. The systems detected as problematic by these checks are recalculated from DFT with improved convergence settings in an attempt to solve the problem (see next next section for more details).

### Compound selection and generation of piezoelectricity data

In this work, we present the piezoelectric tensor for a total of 941 compounds. The compounds were selected from the MP database, with certain constraints applied. These constraints are chosen to specifically target compounds that have the possibility of exhibiting piezoelectric behavior, as follows: 1) only structures with space groups 1, 3–9, 16–46, 75–82, 89–122, 143–146, 149–161, 168–174, 177–190, 195–199, 207–220 are allowed (since these space groups lack inversion symmetry), 2) the calculated DFT bandgap >0.1 eV, 3) the energy above the convex hull (decomposition energy^56^) <0.10 eV/atom, and 4) the number of atoms in the unit cell ≤20. These constraints are chosen to identify a set of materials that has the possibility of exhibiting piezoelectric behavior, while being energetically stable or near-stable and having relatively small unit cells. In particular, materials can only exhibit piezoelectric behavior if they lack inversion symmetry and have an electronic bandgap (constraints 1 and 2, respectively). The space group was determined based on the relaxed structures from the MP database.

For these select compounds, the relaxed structures are extracted from the MP-database and used as input for the DFPT-calculation of the piezoelectric constants. However, DFT convergence parameters chosen for structure relaxations, such as the kpoint-density and the plane wave energy cutoff that are optimized for the total energy, are not in general sufficient for the purpose of calculating properties from DFPT, such as phonons and piezoelectric constants. Hence, the DFPT-calculations are performed using more stringent convergence parameters, as required by the Berry-phase approach^57,58^.

### Workflow

**Figure 4:**
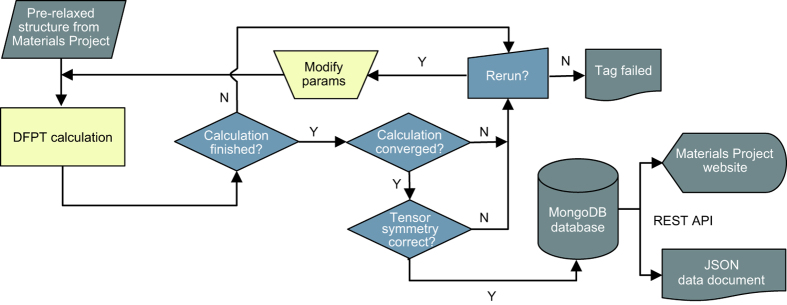
High-Throughput calculation scheme. Flowchart showing a schematic of the HT-infrastructure for calculating piezoelectric constants, including error-checking steps and database insertions.

Figure 4 illustrates the scheme used for the HT-calculation of the piezoelectric constants from DFT. For each selected structure from the MP database, a DFPT-calculation is first carried out, which directly results in the piezoelectric tensor. To ensure reliable calculated constants, we have devised several consistency checks and filters as part of our workflow. The aim is to detect possible errors in the DFT-calculations and other problems such as unconverged results. These filters largely rely on symmetry considerations. Certain classes of point groups impose constraints on the piezoelectric tensor, such as components being identically equal to zero, or components being equal to each other. For example, within the cubic crystal system, either all piezoelectric constants are equal to zero (for non-piezoelectric cubic compounds) or there is only one independent nonzero piezoelectric constant. An overview of the symmetry constraints for the point groups considered in this work is given in Figs 2 and 3.

Our filters take the symmetry considerations above into account and are chosen as follows: (i) $\left|e_{ij}\right|<0.01\;C/m^{2}$ for components that should be identically zero by symmetry (ii) if the point group symmetry imposes that *e*_*ij*_ and *e*_*kj*_ are identical, these should be within 5% in the DFT-calculation and (iii) ${\left\|e_{ij}\right\|}_{\max}<5\;C/m^{2}$. Conditions (i) and (ii) are simply employed to check if the symmetry of the crystal structure is approximately represented in the calculated piezoelectric tensor. The notation ${\left\|e_{ij}\right\|}_{\max}$ in filter (iii) denotes the maximum attainable absolute value of the longitudinal piezoelectric modulus, experienced by the crystal in any direction. For example, as the orientation of the *E*-field with respect to the crystal is varied, the response of the crystal in the direction of the *E*-field can be measured and this can be repeated for all possible directions. ${\left\|e_{ij}\right\|}_{\max}$ corresponds to the maximum longitudinal piezoelectric response that is measured among all directions.

**Figure 5:**
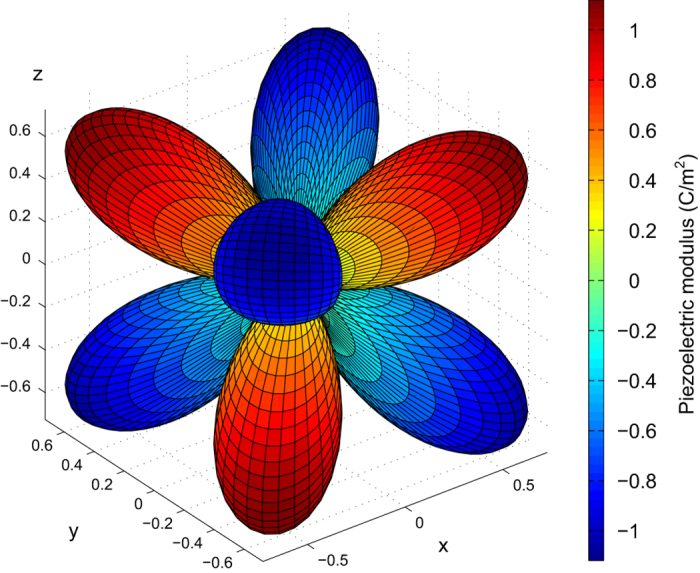
The longitudinal piezoelectric modulus. Visualization of the piezoelectric tensor: directional dependence of the longitudinal piezoelectric constant in cubic LaOF. Note that the maximum and minimum piezoelectric constants, ${\left\|e_{ij}\right\|}_{\max}$, occur for the 〈111〉 family of crystallographic directions.

Figures 2 and 3 provide surfaces with the longitudinal magnitude of the piezoelectric modulus for various crystal symmetries. As an example, for cubic crystals the maximum longitudinal piezoelectric modulus ${\left\|e_{ij}\right\|}_{\max}$ occurs in the 〈111〉 family of directions, as indicated in Figs 2 and 3 and also shown in Fig. 5 for the specific case of cubic LaOF. These directions where ${\left\|e_{ij}\right\|}_{\max}$ occurs are indicated in this work by *v*_max_. The reason for including filter (iii) involving ${\left\|e_{ij}\right\|}_{\max}$ is that the most potent class of currently known piezoelectric materials, lead zirconate titanate (PZT’s), exhibit maximum absolute piezoelectric tensor components in the range of approximately 6–12 C/*m*^2^ (refs 13,59–61), the precise values depending on the details such as grain size, temperature etc. Calculations that yield values in that range are not necessarily wrong, but deserve additional attention due to the relatively large magnitudes.

For materials that do not pass filters (i)-(iii) an additional DFPT-calculation is performed with more stringent convergence parameters. This is similar to our approach taken in recent HT DFT-calculations of elastic constants^34^. If the filters are still not passed after this calculation, a warning tag is attached to that specific compound. In some cases, when the initial DFPT-calculation fails, it is also rerun with more stringent parameters in an attempt to converge the calculation. If it fails a second time, a tag indicating a failed calculation is attached. From the initial set of 941 materials, it is found that 134 materials are flagged by one or more of the filters (i)-(iii). In particular, for 123 compounds we find that filters (i) and/or (ii) are violated, indicating problems with the symmetry of the piezoelectric tensor. In addition, of the 941 compounds considered, 11 systems exhibit a piezoelectric modulus ${\left\|e_{ij}\right\|}_{\max}<5\;C/m^{2}$ and therefore are flagged by filter (iii). For these systems, we repeat the DFPT-calculation of the piezoelectric tensor with an increased k-point density. It is found that this reduces the number of systems that do not pass the filters from 123 down to 81. For the remaining compounds, a warning message is generated on the MP website.

Each piezoelectric tensor that passes the filters is symmetrized according to the point group-symmetry and subsequently inserted into the MP database, see Fig. 4. It is also reported on the MP website. Furthermore, all currently available data can be downloaded from the Dryad-repository (Data Citation 1). There, it is available as a JSON (JavaScript Object Notation) data document. The data analysis is performed using our open-source materials analysis code pymatgen^62^. The HT calculations are automated using the FireWorks workflow software^63,64^.

### Code availability

The proprietary VASP-code is used in this work for the calculation of piezoelectric constants. The filters and symmetrization and analysis code for the piezoelectric constants are implemented in pymatgen^62^. Pymatgen is released under the MIT (Massachusetts Institute of Technology) License and is freely accessible. Further, the open-source code MTEX^65–68^ is used to generate the 3D-representation of the piezoelectric tensors. This code operates on most versions of the widely used MATLAB-software package. The workflow depicted in Fig. 4 is implemented in FireWorks, which is released free of charge under a modified GPL (GNU General Public License).

## Data Records

The calculated piezoelectricity data and the associated metadata of all 941 materials are freely available at the website of the Materials Project (www.materialsproject.org), and the Materials API^69^ can be used to download the data. The complete set of data is also available as a JSON file and can be found in the Dryad-repository (Data Citation 1). It is possible to query materials with certain piezoelectric properties on the MP website via a dedicated web interface. The MP website also includes dedicated details pages for each compound, giving an overview of its calculated properties to date as well as the calculation parameters.

### File format

Metadata is associated with each material and contains information regarding some of the properties of the material, such as crystal structure (space groups, point groups), a unique MP-ID for structure identification and several DFT calculation parameters such as k-point density. The data for each of the calculated materials is stored as a JSON document (Data Citation 1). The JSON format is comprised of hierarchical key-value pairs. Table 1 lists for each of these properties the JSON key, datatype and a short description. To retain consistency with the database of elastic constants, we present the structure of each piezoelectric compound in two ways: (i) Crystallographic Information File (cif) and (ii) poscar-format. The poscar-format is the structure-description as used in the VASP-code and this can be converted into other formats using pymatgen.

### Properties

The piezoelectric tensor reported in this work corresponds to a single crystal. For the elastic constants, polycrystalline isotropic averages of the bulk and shear constants can be derived from the single crystal 4th-order elastic tensor^35,70,71^. For the 3rd-order piezoelectric tensor, however, an isotropic averaging scheme is not convenient, as it will yield isotropic averages equal to zero^7^. Hence in this work, we report the piezoelectric tensor in matrix form, together with several properties that are expected to be of use to the community, see Table 2. The piezoelectric tensor *e*_*ij*_ as presented in this work (see Table 2) is expressed in the standardized IEEE-format^72^, corresponding to the notation as shown in Figs 2 and 3. Note that the symmetrized piezoelectric tensors are presented in this work, obeying the point group symmetry of each of the compounds. We further report ${\left\|e_{ij}\right\|}_{\max}$, which was defined earlier as the maximum longitudinal piezoelectric modulus of the compound in any crystallographic direction (see Fig. 5). The associated crystallographic direction is also reported, which corresponds to the direction of the *E*-field that leads to the maximum normal stress in that same direction. Finally, the symmetry information of each compound is included (space and point groups) since these are closely related to piezoelectric properties.

### Graphical representation of results

**Figure 6:**
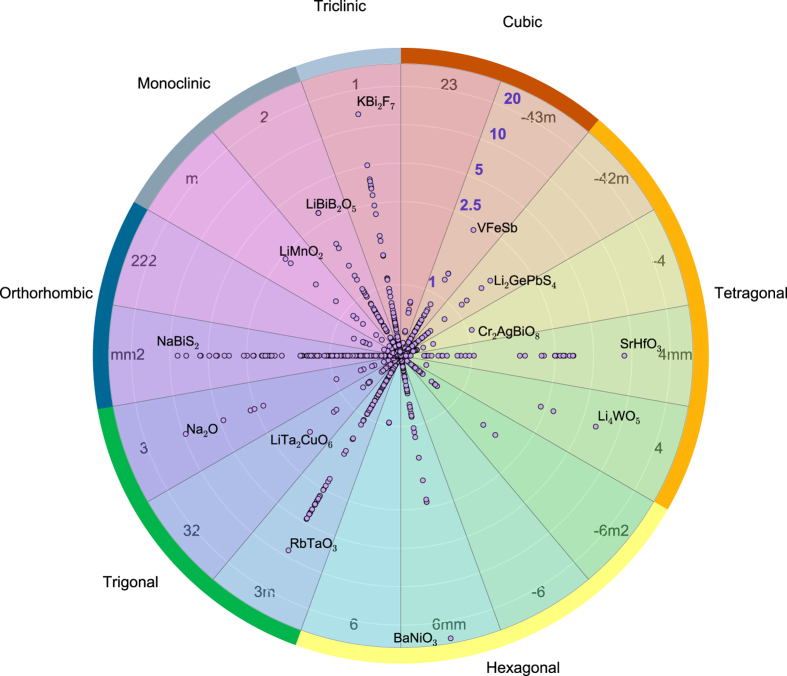
Distribution of piezoelectric constants. A graphical representation of the piezoelectric dataset, currently comprising of 941 materials. A series of concentric circles indicate constant values of the maximum longitudinal piezoelectric modulus, ${\left\|e_{ij}\right\|}_{\max}$. Concentric circles corresponding to moduli ${\left\|e_{ij}\right\|}_{\max}$ of 1, 2.5, 5, 10 and 20 C/m^2^ are indicated explicitly in the figure. The compounds are broken up according to the crystal system and the different point group symmetry-classes considered in this work.

Our dataset is presented graphically in Fig. 6, where the maximum piezoelectric modulus ${\left\|e_{ij}\right\|}_{\max}$ for 941 compounds is plotted in a pie-chart, which also shows the point group symmetry-classes considered in this work (see also Figs 2 and 3). The results in Fig. 6 are broken up by the 7 crystal systems, which are further subdivided into the point groups that can exhibit piezoelectric behavior.

From Fig. 6 it follows that a large fraction of the compounds are located near the center of the chart, which indicates relatively weak piezoelectric behavior $\left(≲1\;C/m^{2}\right)$ of ${\left\|e_{ij}\right\|}_{\max}$. On the other hand, we find that 17% of the compounds in the dataset satisfy ${\left\|e_{ij}\right\|}_{\max}\geq 1\;C/m^{2}$ and 5% satisfies ${\left\|e_{ij}\right\|}_{\max}\geq 3$, indicating relatively large piezoelectric behavior. Our HT-calculations confirm high (intrinsic) piezoelectric constants for compounds such as PbTiO_3_, BaNiO_3_, RbTaO_3_ and SrHfO_3_, some of which are indicated in Fig. 6. We further identify a set of potent piezoelectric compounds that (to the best of our knowledge) have not yet been confirmed experimentally or computationally in the literature. Examples are VFeSb, Li_4_WO_5_, LiMnO_2_, NaBiS_2_ and a few dozen others that are present in Fig. 6, but not explicitly indicated. The identification of these compounds, exhibiting interesting piezoelectric behavior, can hopefully contribute to the search for novel new piezoelectric materials. Of particular interest is also how different synthesis techniques can affect the intrinsic piezoelectric response, as shown in Fig. 6, by changing for example the grain size, the impurity concentration, and by introducing defects. In fact, the most widely-used piezoelectric compounds today are based on lead zirconate titanate (PZT’s). Their high piezoelectric response stems from a careful tuning of the checmical composition to a region that is near a morphotropic phase boundary^73–76^. In this work, the effect on the piezoelectric properties of alloying to create solid solutions is not considered, however, the intrinsic piezoelectric moduli such as shown in Fig. 6 may provide a convenient starting point in the process of searching for new piezoelectrics.

## Technical Validation and Verification

### Verification of computational methodology

Verification of the current HT implementation for calculating piezoelectric constants by the DFPT Berry phase-approach is undertaken through comparison of the present results to those obtained in the literature. Comparisons are made with work in the literature using similar DFT-methods but alternative implementations of DFPT or a finite strain-based method rather than DFPT. As an example, for the wurtzite-compound AlN, we calculate *e*_33_=1.46 C/*m*^2^ and *e*_31_=−0.58 C/*m*^2^ which is within 10% of the values reported elsewhere, obtained using GGA as well, but with a different implementation of DFPT and slightly different convergence parameters^77^. Similar or better levels of agreement are obtained for the compounds GaN and InN^77^ and several (wurtzite) oxides with piezoelectric behavior such as BeO and ZnO^8^. For the ternary oxide PbTiO_3_, we compare our calculated piezoelectric constants to those calculated from DFPT and a finite strain method^13^. For all 3 components of the piezoelectric tensor, we find agreement to within 15% for both the DFPT and finite strain-calculations. We further compare our calculated piezoelectric constants to those reported in the literature^78^ for BiAlO_3_. We find that for the constants *e*_31_, *e*_33_ and *e*_15_, the agreement is within 15–20%. For the smallest modulus of the tensor, *e*_21_, the agreement is worse, as it differs by approximately a factor 2.5, however the absolute difference is similar to that found for the other components. Even though the method employed in^78^ is also based on DFPT and uses GGA-PBE, it employs a Troullier-Martins norm-conserving pseudopotential methodology^79^ to describe electron-ion interaction, which differs from the PAW method used in the current approach. We expect that the discrepancy in *e*_21_ may be caused by this difference.

Based on the comparisons performed as part of this work, we find that the level of agreement between the piezoelectric constants calculated from our HT-methodology and those obtained in the literature using alternative methods, generally show agreement to within approximately 15–20%. This level of agreement is consistent with that found in the literature, comparing piezoelectric constants calculated from DFT (e.g., refs 8,80).

### Validation through comparison to experimental measurements

In order to gauge the expected accuracy of the calculated results, an extensive comparison with reported experimental piezoelectric constants was performed. To achieve consistency with the calculations, we consider comparisons only with measurements that report the complete piezoelectric tensor, rather than just a subset of components. This leads to a comparison for 36 systems, and over 75 independent piezoelectric tensor components. The systems for which data from the literature is taken range from well-known semiconductors such as GaAs and InAs to binary compounds such as ZnO, ZnTe and oxides of the form XYO_3_ or XYO_2_ such as SnTiO_3_, LiNbO_3_ and LiGaO_2_.

**Figure 7:**
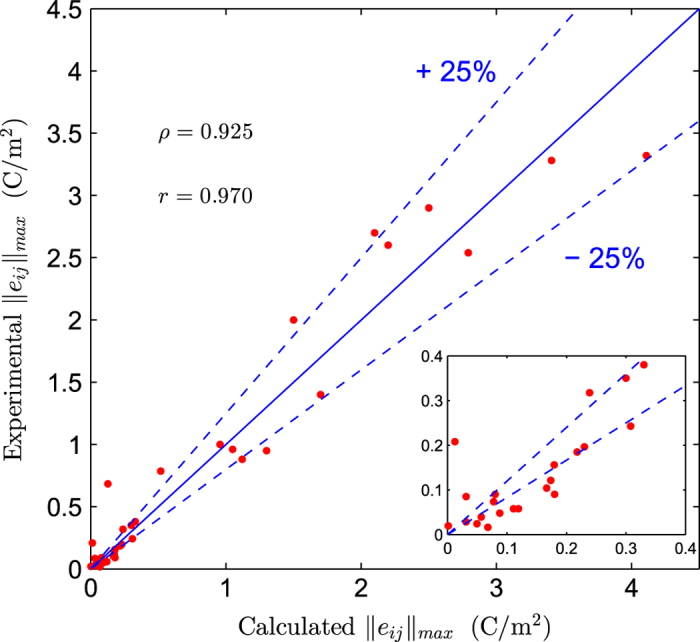
Plot of experimental versus calculated piezoelectric constants. Comparison of experimental and calculated piezoelectric constants $\left({\left\|e_{ij}\right\|}_{\max}\right)$ for a selected set of systems, with calculated Pearson correlation coefficient *r* and Spearman correlation coefficient *ρ* reported.

The comparison of calculated and experimental values for the piezoelectric constants are shown in Fig. 7. The points represent the quantity ${\left\|e_{ij}\right\|}_{\max}$, which represent the maximum attainable piezoelectric response (over all crystallographic directions) and is derived directly from the calculated and experimentally determined piezoelectric tensors. In the plot, lines are shown indicating relative differences between computation and experiment of ±25%. A threshold of 25% is chosen since this represents a typical discrepancy between experiment and calculation for the case of piezoelectric constants. Note that this is true in particular for compounds with relatively large piezoelectric constants. The inset of Fig. 7 shows that for values below roughly 0.4 C/m^2^, percentage errors are much larger. The same trend was observed in our recent work on elastic constants^34^, although for piezoelectric constants, the discrepancies between our DFT-calculations and experiments tend to be larger. Discrepancies between experiment and calculation of over 25% are identified for 16 systems, which are (in order from high to low discrepancies): ZnS, GaP, InP, BeO, BP, CdTe, InAs, SiBiO, InSb, GaSb, AlSb, GaAs, CdS, BN, AlN and CuCl.

There are several other factors that can contribute to discrepancies between calculations and the experiments. First of all, our DFT-calculations provide a description of the materials that is strictly only valid at a temperature of 0 K. However, most experiments are done at room temperature or elevated temperatures. Temperature can have a significant effect on the measured piezoelectric response of materials. For example, for lead zirconate, the absolute values of the piezoelectric constants were reported to increase by as much as 46% as temperature increases from −55 C° to 85C° (ref. 81). Similar temperature dependence was found in other work for lead titanate^82^. In addition to temperature effects, it should be noted that our calculations probe only the intrinsic contribution to the piezoelectric behavior of materials, assuming a perfectly ordered bulk crystal. Hence, extrinsic effects associated with defects and grain size are not considered; grain size is known to influence piezoelectric properties in some compounds such as BaTiO_3_ (ref. 83).

We further note that piezoelectric coefficients can be strongly affected by variations in lattice constants. This has been established in the literature based on detailed investigations for a number of systems^84–86^. For example, in some piezoelectric compounds such as PbTiO_3_ and BaTiO_3_, hydrostatic pressures of less than 1 GPa can lead to variations in the piezoelectric tensor components of up to 50% (refs 87,88). We observed a similar effect where the choice of either the LDA or GGA approximation to the exchange-correlation energy led to differences in the predicted lattice constants by 1–2%, with an associated change in the piezoelectric constants of as much as 40%. In this work, GGA-PBE is used for the piezoelectric calculations to obtain consistency with other data tabulated in the Materials Project. We further found that for compounds containing specific elements, different piezoelectric constants were obtained, depending on how many electrons were used as valence states. This is especially true for the early transition metals such as Sc, Ti, V and Nb, for which piezoelectric components can differ by up to several percent, depending on the details of the PAW-potential. Consistent with the framework of MP, for elements such as these, semi-core states are included in the calculations.

In order to obtain a statistical measure of how well the measured and calculated piezoelectric constants, ${\left\|e_{ij}\right\|}_{\max}$, agree, we compute the Pearson (*r*) and Spearman (*ρ*) correlation coefficients. These quantities provide insight into how strongly the measured and calculated piezoelectric constants are linearly associated and monotonely associated, respectively. We find that *ρ*=0.925 and *r*=0.970. This implies that the measured and calculated values for the maximum longitudinal piezoelectric constants exhibit a strong linear association and also, a high monotone association exists. This makes the database particularly useful for screening and datamining of structure-chemistry correlations in piezoelectrics.

## Usage Notes

In this work, we present a database of calculated intrinsic piezoelectric constants for 941 inorganic crystalline compounds, for use in the design and development of piezoelectric materials and devices. Specifically, we expect this database to enable searches for new, previously unknown, piezoelectric materials or as an aid in screening for replacement candidates for currently known interesting piezoelectric materials such as PbTiO_3_. Our database allows researchers to search through a large pool of compounds and select those with certain target piezoelectric responses, for example a tensile strain larger than some threshold value upon the application of a given electric field. In addition, researchers can query the database and screen for materials obeying ‘composite constraints’, for example a combination of desired piezoelectric response, elastic properties and mass density. These features are expected to be of interest to researchers working in a variety of fields, such as piezoelectricity, elasticity and thermodynamic properties. Similar to the previous work on elastic constants, as part of the future work on piezoelectric constants, a web interface will be implemented in which MP-users can request calculated piezoelectric constants for compounds that have not been considered yet up to now. Other possible future work includes the use of statistical methods such as machine learning on the current database to better understand the structure-chemistry descriptors that give rise to interesting behavior (e.g., high piezoelectric constants). Eventually, techniques such as those may assist the accelerated search for new materials with unprecedented piezoelectric properties.

## Additional Information

**How to cs article:** de Jong, M. *et al.* A database to enable discovery and design of piezoelectric materials. *Sci. Data* 2:150053 doi: 10.1038/sdata.2015.53 (2015).

## Supplementary Material



## Figures and Tables

**Table 1:** JSON keys for metadata and their descriptions.

**Key**	**Datatype**	**Description**
material_id	string	IDs for entries in the Materials Project
formula	string	Chemical formula
structure	string	Relaxed crystal structure represented in Crystallographic Information File (cif)
poscar	string	relaxed crystal structure represented in poscar-format for VASP calculations
space_group	number	Space group number defined by The International Union of Crystallography
point_group	string	Point group in Hermann-Mauguin notation
volume	number	Volume of the relaxed structure in Å^3^
nsites	number	Number of atomic sites for the conventional cell
kpoint_density	number	density of k-points in the first Brillouin zone per reciprocal atom

**Table 2:** Properties related to the piezoelectric tensor in this work, and their corresponding JSON keys and datatypes.

**Property**	**Key**	**Datatype**	**Unit**	**Description**
Piezoelectric tensor, *e*_*ij*_	piezoelectric_tensor	array	C/m^2^	Tensor, describing piezoelectric behavior (IEEE-format)
Piezoelectric modulus, ${\left\|e_{ij}\right\|}_{\max}$	eij_max	number	C/m^2^	Maximum longitudinal piezoelectric modulus
Crystallographic direction, *v*_max_	v_max	array	—	Crystallographic direction, corresponding to maximum piezoelectric modulus

## References

[d1] Dryad De Jong M. Chen W. Geerlings H. Asta M. Persson K. 2015 http://dx.doi.org/10.5061/dryad.n63m4 10.1038/sdata.2015.53 PMC4587372 26451252

